# Analysis of the misdiagnosis of 8 adult cases of paragonimiasis with lung masses as the main manifestation in Xishuangbanna, Yunnan

**DOI:** 10.1186/s13019-021-01408-y

**Published:** 2021-03-19

**Authors:** Qiu-Hong Shu, Yang Yang, Shu-De Li, Jun-Sheng Zhao, Sheng-Hao Li, Miao-Miao Wang, Wei-Qun Wang, Ming Tian, Shu-Mei-Qi He, Zhi-Qiang Ma, Min Zhu, Wen-Lin Wang

**Affiliations:** 1 The Second Affiliated Hospital of Kunming Medical University, No. 374, Dianmian Road, Kunming, 651010 Yunnan China; 2 Oncology Department, People’s hospital of Xishuangbanna Dai Autonomous Prefecture, Xishuangbanna, Jinghong, Yunnan China; 3 Kunming Medical University, No.1168, Chunrong West Road, Yuhua Street, Chenggong District, Kunming, 65050 Yunnan China; 4 Mengma Town Central Health Center, Menglian, Pu’er City, Yunnan China; 5 The Third People’s Hospital of Kunming, Kunming, China; 6 The 2nd People’s Hospital of Chengdu, No.10, Qingyun South Road, Jinjiang District, Chengdu, 510104 Sichuan Province China

**Keywords:** Paragonimiasis, Misdiagnosis, Lung masses

## Abstract

**Objective:**

To summarize the clinical characteristics of adult cases of paragonimiasis with lung masses as the main manifestation in Xishuangbanna, Yunnan Province, analyze the causes of misdiagnosis, and improve the levels of clinical diagnosis and treatment.

**Method:**

We conducted a retrospective analysis of the clinical data and diagnosis and treatment of 8 adult cases of paragonimiasis with lung masses as the main manifestation that were diagnosed in the Oncology Department of People’s hospital of Xishuangbanna Dai Autonomous Prefecture from July 2014 to July 2019.

**Result:**

All 8 patients were from epidemic paragonimiasis areas and had a confirmed history of consuming uncooked freshwater crabs. The clinical manifestations were mainly fever, dry cough, and chest pain. The disease durations were long, and peripheral blood eosinophil counts were elevated. The cases had been misdiagnosed as pneumonia or pulmonary tuberculosis. After years of anti-inflammatory or anti-tuberculosis treatment, the symptoms had not improved significantly. Patients eventually sought treatment from the oncology department for hemoptysis. Chest computed tomography showed patchy consolidation in the lungs, with nodules, lung masses, and enlarged mediastinal lymph nodes.

**Conclusion:**

Paragonimiasis is a food-borne parasitic disease. Early clinical manifestations and auxiliary examination results are nonspecific. The parasite most often invades the lungs, and the resulting disease is often misdiagnosed as pneumonia, pulmonary tuberculosis, or lung cancer (Acta Trop 199: 05074, 2019). To avoid misdiagnosis, clinicians should inquire, in detail, about residence history and history of unclean food and exposure to infected water and make an early diagnosis based on the inquired information and imaging examination results. For patients who have been diagnosed with pneumonia or pulmonary tuberculosis and whose symptoms do not improve significantly after anti-inflammatory or anti-tuberculosis treatments, their epidemiological history should be traced to further conduct differential diagnosis and avoid misdiagnosis.

## Introduction

Paragonimiasis is a systemic chronic parasitic disease caused by *Paragonimus* infection. It is a natural focal disease and one of the food-borne parasitic diseases that cause great harm to both humans and animals [1]. The onset of the disease is insidious, and there is currently a lack of clinically specific diagnostic methods [2]. Medical staff have insufficient knowledge of the disease, and the misdiagnosis rate is high. Paragonimiasis in adults is often misdiagnosed as pneumonia, pulmonary tuberculosis, or even lung cancer [3]. Here, we summarize the clinical manifestations and causes of misdiagnosis of 8 adult cases of paragonimiasis with lung masses as the main manifestation in People’s hospital of Xishuangbanna Dai Autonomous Prefecture in the past 5 years, so as to improve the understanding of the disease among medical professionals and reduce the misdiagnosis rate.

## Materials and methods

### General information

Eight patients with paragonimiasis with lung masses as the main manifestation who were hospitalized and diagnosed in People’s hospital of Xishuangbanna Dai Autonomous Prefecture, from July 2014 to July 2019 were enrolled as the clinical research subjects. There were 5 males and 3 females, aged 49–61 years old, with an average age of 52 years. Among the 8 cases, 6 were misdiagnosed as pneumonia, and 2 were misdiagnosed as pulmonary tuberculosis. The patients received standardized anti-inflammatory or anti-tuberculosis treatment. In the early stage of treatment, the symptoms were slightly alleviated but without significant alleviation. After completing a course of treatment, the symptoms returned or even worsened, and patients then sought treatment again. Computed tomography (CT) of the lungs revealed a lung mass, and the patients were admitted to the oncology department for further treatment.

### Method

For all of the abovementioned patients, we recorded the life history, the main clinical manifestations, the laboratory findings and imaging examination results and clinical diagnosis and treatment history and conducted a retrospective analysis.

### Genomic analysis of the parasites identified during surgery

Based on the ITS2 sequence obtained in GenBank, Premier (5.0) software was used to design primers: 3S’: 5′-GGTAC CGGTG GATCA CTCGG CTCGT G-3′ and A28: 5′-GGGAT CCTGG TTGT TTCTCTT CCGC-3′. The bodies of the worms were washed 3 times with 0.9% normal saline (NS), placed into 20 μl of digestion solution (3 mmol/l MgCl_2_, 100 mmol/L KCl, 20 mmol/L Tris-HCl, pH 8.5, 0.9% NP-40, 0.02% gelatin, 800 mg/ml proteinase K and 0.9% Tween-20), and incubated at 55 °C for 2 to 4 h. Then, the proteinase K was inactivated by heating the digestion solution at 95 °C for 4 min. A total of 1 μl of the digestion solution containing the genomic DNA of the worms was used to carry out polymerase chain reaction (PCR) in 25 μl of PCR buffer. The reaction system contained 1 μl of deoxynucleoside triphosphates (dNTPs), 1 μl of forward primer 3S’, 1 μl of reverse primer A28, 1 μl of bovine serum albumin (BSA) and 0.15 μl of Tap DNA polymerase (Promega). After denaturation (95 °C) and annealing (1 min), Elongation (1 min) was performed. The Elongation was completed at a low temperature. To ensure the accuracy of the sequencing, positive and negative strand sequencing was performed on the PCR products generated by each primer combination. The obtained sequences were compared for homology using the National Center for Biotechnology Information (NCBI) BLAST.

## Results

### Life history of the patients

Among the 8 patients, 2 were of Han ethnicity, and 6 were of ethnic minorities. They all lived in epidemic areas and caught crabs in streams to make pickled crab sauce. All 8 patients had eaten raw crab sauce.

### Clinical manifestations

The main symptoms of the patients were respiratory symptoms: 3 patients had a chronic dry cough, 2 patients had chest pain, 1 patient had fever, and 2 patients had hemoptysis during the course of chronic cough and wasting. The disease course was long. All 8 patients had received anti-inflammatory and anti-tuberculosis treatments. The symptoms repeatedly occurred without any obvious improvement. The patients were in poor spirits, had normal bowel movements, and experienced significant weight loss.

### Laboratory examination

Total leukocyte counts and eosinophil-to-lymphocyte ratios were significantly increased based on complete blood counts. C-reactive protein levels were increased, and erythrocyte sedimentation rates were increased significantly. Total immunoglobulin G (IgG) and immunoglobulin M (IgM) levels in all patients were significantly increased, and all patients had IgG > 20 g/l. The tuberculin tests of 5 patients were positive; these results were considered to be related to tuberculosis vaccination. After the diagnosis of paragonimiasis in the later period, serum samples from the patients were used for enzyme-linked immunosorbent assays to detect serum antibodies to *Paragonimus*; 6 patients were positive. The summary are as show in Table 1.

**Table 1:** The characteristics of the eight patients

NO.	Gender	Age (Y)	MCM	Laboratory Findings							
				TLC 4–10 (× 10^9^/L)	ELR 0.8–5.3(%)	CRP 0–10 (mg/L)	ESR 0–20 (mm/s)	IgG 7–16 (g/L)	IgM 0.7–2.3(g/L)	TBT	SAP
1	F	57	chronic dry cough	13.48	9.11	17.1	28	26.3	3.0	–	+
2	F	59	fever	20.1	8.00	18.89	41	22.07	3.97	–	–
3	F	61	Hemop-tysis	18.04	12.01	16.05	27.4	25.1	3.44	+	+
4	M	49	chronic dry cough	19.19	7.89	22.0	33	23.64	4.01	+	+
5	M	50	chronic dry cough	17.65	9.96	15.08	52	27.6	3.62	+	+
6	M	51	chest pain	12.02	9.22	16.66	46	25.09	4.98	+	–
7	M	57	chest pain	16.22	8.03	18.9	32	22.13	4.32	–	+
8	M	60	Hemop-tysis	14.21	7.8	16.21	27.9	24.35	4.09	+	+

*MCM* Main clinical manifestations; *TLC* Total leukocyte counts; *ELR* Eosinophil-to-lymphocyte ratios; *CRP* C-reactive protein levels; *ESR* Erythrocyte sedimentation rates; *IgG* immunoglobulin G; *IgM* Immunoglobulin M; *TBT* Tuberculin test; *SAP* enzyme-linked immunosorbent assays to detect serum antibodies to *Paragonimus*

### Imaging

**Fig. 1:**
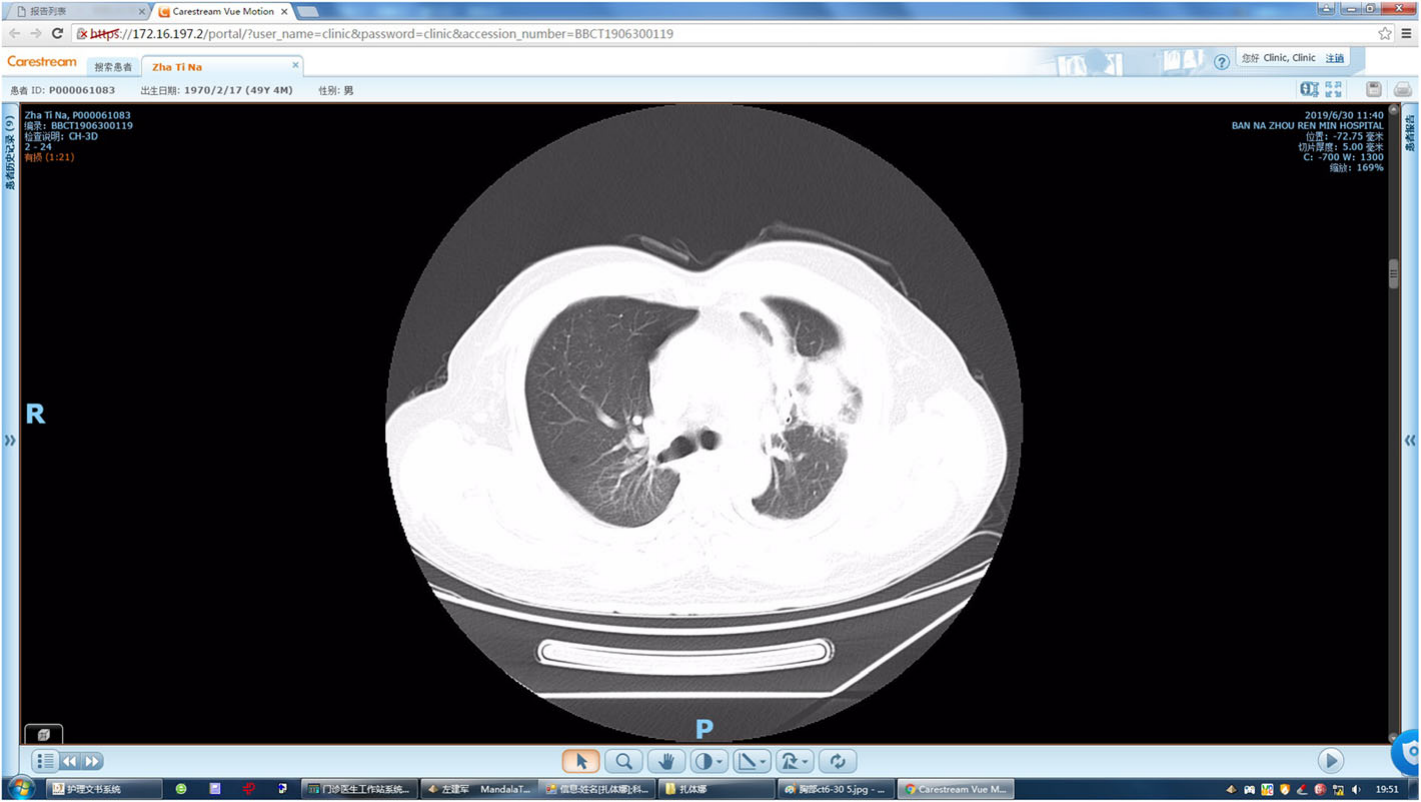
Irregular lobulated mass in anterior segment of left upper lobe, peripheral floccule inflammation, djacent pleural thickening adhesion, local traction

Chest CT showed a symmetrical thoracic cage, no displacement of the mediastinum or trachea, and an increased number of lung markings bilaterally. Lung masses were seen in 8 patients, with unclear borders and uneven attenuation. Two patients had small cavities in the masses, 3 patients had enlarged lymph nodes in the mediastinum, and 5 patients had a small amount of pleural effusion in the thoracic cavity on the same side as the lesion. Typical signs are as shown in Fig. 1.

### Diagnosis and treatment process

The 8 patients were treated at local hospitals for mild pulmonary symptoms in the early stage of disease onset. Based on the previous experience of the physicians, pulmonary inflammation was considered. All 8 patients had received anti-inflammatory treatment before admission. The drugs included cephalosporins and/or quinolones. In the early stage of anti-inflammatory treatment, symptoms were slightly relieved. After receiving a course of treatment, the symptoms were not significantly alleviated. The patients returned to the hospital for subsequent examinations. According to the chest CT results, 6 patients were diagnosed with pneumonia, and their antibiotic treatment was adjusted; 2 patients were diagnosed with pulmonary tuberculosis and were given anti-tuberculosis treatment. However, the treatment effect was still poor, cough symptoms worsened, and the patients developed poor spirits and exhibited weight loss. Two of the patients developed hemoptysis. The patients then went to People’s hospital of Xishuangbanna Dai Autonomous Prefecture for treatment. Lung CT revealed lung masses.

To clarify the cause of the lung masses, the patients were admitted into the oncology department, and thoracoscopic resection of the lung masses was performed. An anterior segmentectomy of the middle lobe of the right lung is used as an example, and the surgical procedure is as follows: after successful anesthesia, the left lateral decubitus position is taken, the right side is up, the seventh intercostal space on the right axillary midline is taken as the observation hole, the fourth intercostal space on the right axillary front line is about 3 cm in length, and the main operation hole is seen. Intraoperatively, closed chest, multiple adhesion bands on the thoracic cavity, separation of adhesion, routine free pulmonary ligament, free separation of the inferior lobe vein of the right lung, and separation of the middle lobe veinArtery and trachea, 2 endoscopy using cutting suture and disposable staple lock to open oblique fissure, 3 endoscopy using cutting suture and disposable staple lock to remove the anterior segment of the middle lobe of the right lung, remove the anterior segment of the middle lobe, dissect the mass, expand the lung test water, check for no air leakage and active bleeding, leave chest drainage tube in place, and close the chest layer by layer.

**Fig. 2:**
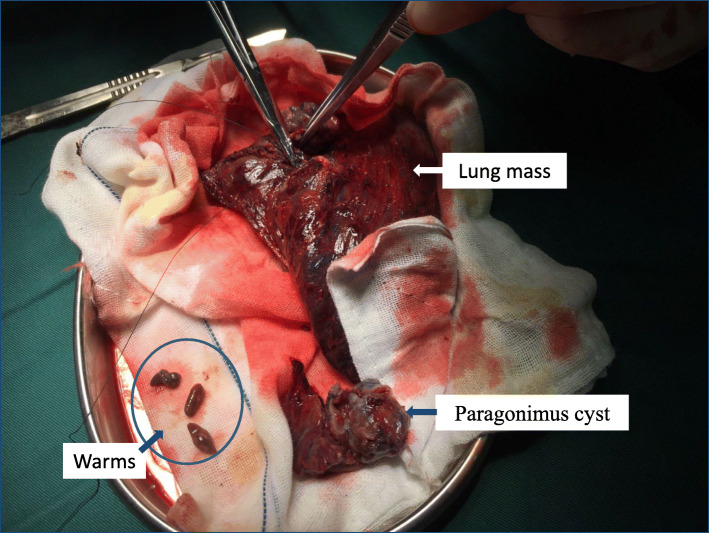
Intraoperative pulmonary mass, paragonimus cyst and worms can be found in the mass

**Fig. 3:**
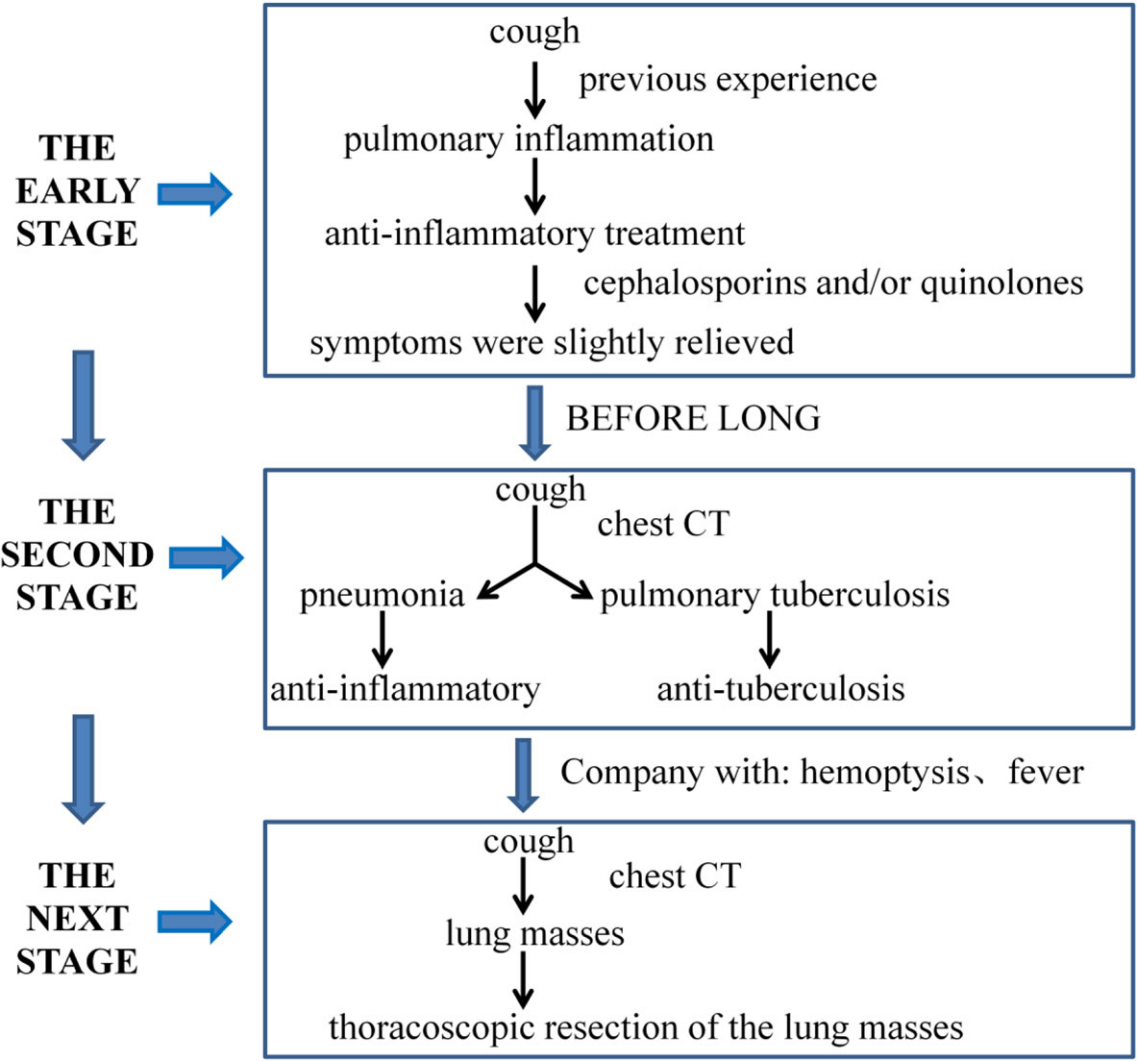
The course of treatment

In 2 patients, *Paragonimus* was found during the operation (Fig. 2). *Paragonimus* bodies were fixed with alcohol and were sent to the Department of Pathogenic Biology, Kunming Medical University, for further testing; the results confirmed the bodies as *Paragonimus heterotremuse*. In the remaining 6 patients, postoperative pathological examination of the masses was performed to exclude lung tumors. Medical histories were again obtained, and parasitic antibody test results were positive, confirming the diagnosis. Praziquantel was given postoperatively for a total of 2 courses, with a total of 210 mg/kg of praziquantel per course, with 3 days as a course of treatment. After praziquantel treatment, blood eosinophil counts gradually decreased and returned to normal in approximately 4 weeks. The symptoms were completely relieved, and chest CT was conducted, indicating the complete absorption of the lung lesions. The course of treatment are as show in Fig. 3.

### Pathogenic examination

#### Morphological examination

**Fig. 4:**
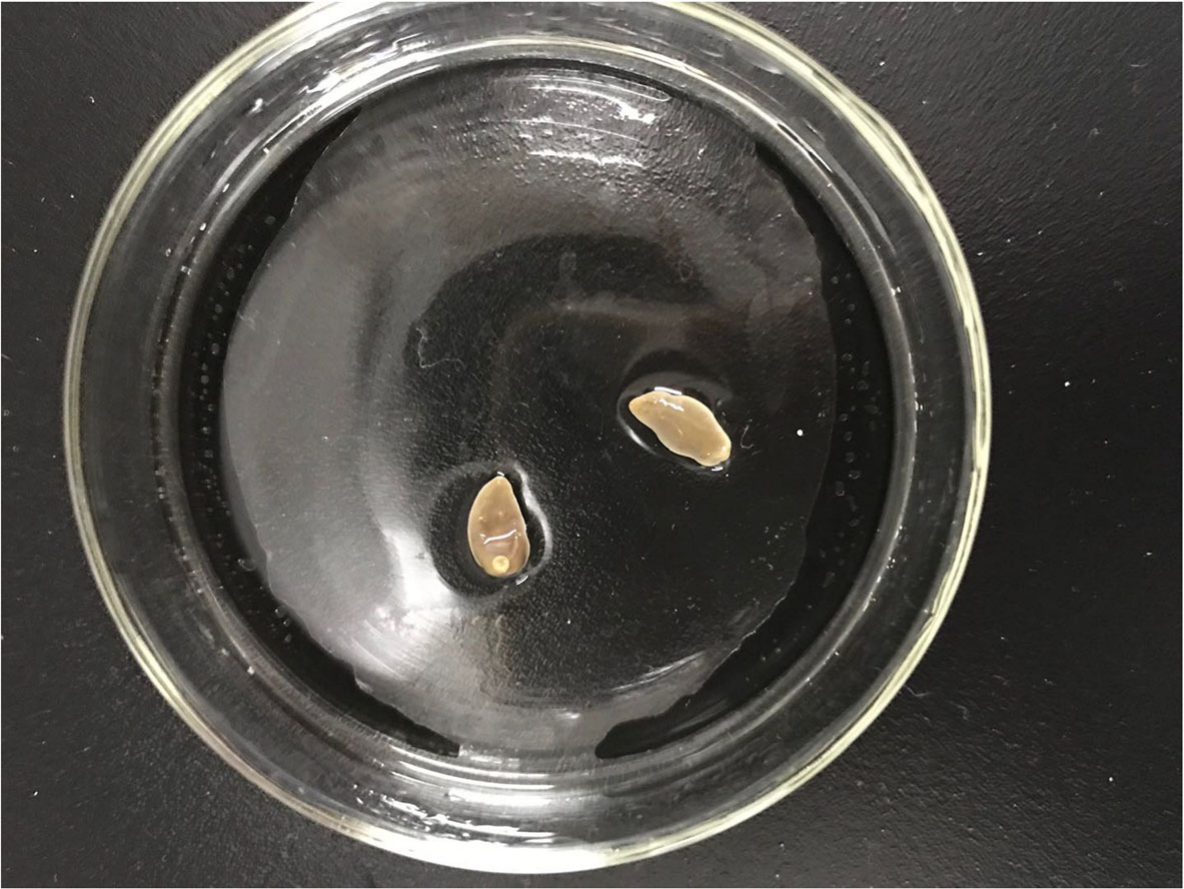
The warms fixed with alcohol

Etiological examination was conducted after the worm bodies found during the operation were fixed in alcohol. Microscopic examination revealed that the worm bodies were thick, the back was slightly convex, the abdomen was flat, the width to length ratio was 1:2, and the uterus was not large and was located between the ventral sucker and the testis, suggesting *Paragonimus heterotremuse* (Fig. 4).

#### Genomics examination

**Fig. 5:**
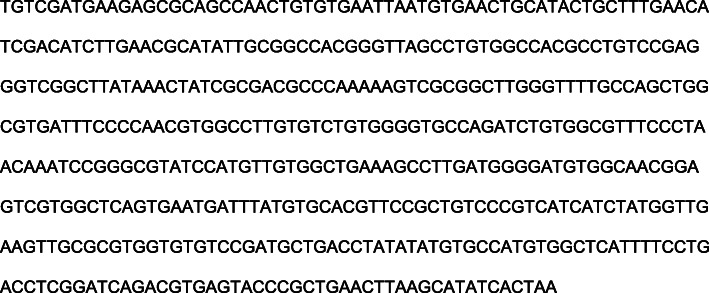
An aligned nucleotide sequence of ITS2 region obtained *Paragonimus heterotremus* worm from the patient by PCR amplification

**Fig. 6:**
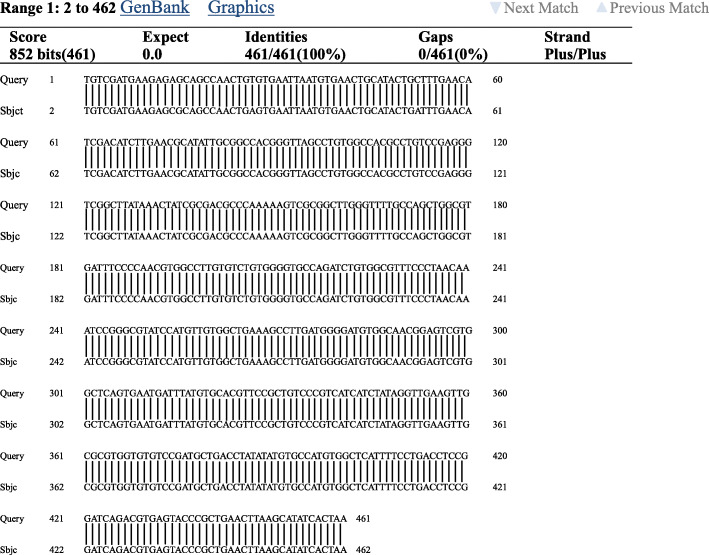
Alignment of ITS2gene sequence of *Paragonimus heterotremus* eggs from patient in Yunnan province,were 100% identical with that of P. heterotremus from the gene bank

The complete ITS2 gene sequence of the worm bodies was analyzed using BLAST-N (NCBI, Bethesda, Maryland); the length of the ITS2 sequence was 461 bp (Fig. 5). The MEGA4 software was used for DNA comparisons, and the ITS2 sequence of the detected parasites were completely consistent with the sequence of *Paragonimus heterotremuse* (GenBank) (Fig. 6), supporting the diagnosis of paragonimiasis.

## Discussion

Paragonimiasis is a systemic chronic parasitic disease. It is a natural focal disease and one of the food-borne parasitic diseases that are harmful to both humans and animals [4]. Paragonimiasis is widely distributed. Blair et al. have shown that the disease is widely distributed in many countries and regions in Asia, Africa, and the Americas, and it is estimated that 292.8 million people in the world are at risk of paragonimiasis [1]. Humans become infected with *Paragonimus* mainly through the consumption of raw or semi-raw freshwater crabs containing metacercaria [3]. The Dai people are the main ethnic minority living in Xishuangbanna. They eat raw freshwater crabs. In Xishuangbanna, the incidence of paragonimiasis is high, but the detection rate is low. The lung is the most common site of *Paragonimus* infection [5]. Due to the nonspecific clinical manifestations of the disease in the early stage, patients often do not go to a hospital until the late stages of disease infection, when the manifest symptoms such as hemoptysis and weight loss. In this study, the 8 patients had thoracic and lung type paragonimiasis.

In this study, the total leukocyte count and the eosinophil-to-lymphocyte ratio in all patients were significantly increased. However, the specificity of eosinophils for the diagnosis of paragonimiasis is not high, and other diseases such as allergic diseases and hematological diseases can also cause the same changes. Therefore, paragonimiasis is often misdiagnosed at the first diagnosis due to the lack of specific diagnostic criteria.

The imaging manifestations of paragonimiasis are consistent with the tissue damage and pathological response caused by the migration of the parasite in the human body. The infection often manifests as unilateral or bilateral patchy infiltrates with uneven densities and blurred edges. The parasites stimulate hyperplasia of the local granulation tissue, and the hyperplastic tissue manifests as single or multiple nodules that vary in size, have clear edges, are mostly located under the pleura, and have internal necrotic low-attenuation areas. The granulation around the nodules gradually develops into fibrosis and forms the cyst wall, leading to single- or multilocular cystic changes and formation of hollow lesions after the cyst contents are discharged [6]. Therefore, nodules, small cavities and multilocular cystic changes are characteristic imaging changes of paragonimiasis [7]. The worms migrate or parasitize in the pleural cavity and lungs. Mechanical damage and worm metabolites can stimulate the pleura, producing effusion, with more pleural effusion on the right side than the left side [8]. The early imaging changes in the 8 patients in this study were similar to those of pneumonia, pulmonary tuberculosis, and lung abscesses, leading to misdiagnosis at the initial diagnosis. As the disease progresses, nodules, masses, and enlarged mediastinal lymph nodes were observed, and the disease can be easily misdiagnosed as a lung tumor. Therefore, chest imaging changes in patients with paragonimiasis are not specific.

The clinical manifestations of and auxiliary examination results for paragonimiasis are similar to common respiratory diseases, leading to misdiagnosis. The current clinical diagnostic methods for paragonimiasis mainly include the examination of *Paragonimus* eggs from sputum and feces; *Paragonimus* intradermal tests and complement fixation tests; immunological examination for antibodies to *Paragonimus*; and the detection of increases in eosinophils [9]. However, the existing diagnostic methods lack specificity, the detection rate is low, and the misdiagnosis rate is high. For most patients, due to the lack of typical clinical manifestations of paragonimiasis and the presence of positive findings on lung imaging examinations often manifesting as the imaging features of lung inflammation, nodules, and masses [10], paragonimiasis is often misdiagnosed as pulmonary tuberculosis or lung cancer, which brings great mental and economic pressure and burden to patients and their families. In this study, horacoscopic resection of the lung masses was performed for the 8 patients. Worms were found intraoperatively in 2 patients. The worms were clearly identified as lung fluke adults by morphological and genetic alignment.

## Conclusion

This study summarized the clinical diagnosis and treatment of 8 patients and put forward the following points for attention to reduce the misdiagnosis and missed diagnosis rates for paragonimiasis. (1) Inquiries regarding medical history should be comprehensive and detailed. All patients in this study had a life history of consuming raw freshwater crabs. Therefore, for patients with lung masses, it is necessary to routinely ask about their birthplace, life history, history of living in epidemic areas, history of eating crabs and crayfish, and exposure to infected water. (2) Paragonimiasis should be considered for patients with elevated eosinophils. In this study, routine blood tests showed that the eosinophil-to-lymphocyte ratio in the patients was increased. All patients with an unexplained increase in eosinophilia should be asked routinely for their epidemiological history, and parasite-related laboratory tests should be performed if necessary. In addition, a decline in or return to normal of eosinophils after treatment can be used as a simple indicator for diagnosis and efficacy evaluations. (3) Paragonimiasis should be considered for patients whose clinical manifestations are not consistent with the usual disease course. In this study, the patients had slow onset, long disease courses, and recurrent symptoms, and the conventional anti-infection and anti-tuberculosis treatments had poor efficacy. (4) For patients with an epidemiological history and lung masses on lung imaging examinations, laboratory tests related to paragonimiasis are required to assist in the diagnosis.

## Data Availability

Not applicable.
